# Residual risk of mother-to-child transmission of HBV despite timely Hepatitis B vaccination: a major challenge to eliminate hepatitis B infection in Cambodia

**DOI:** 10.1186/s12879-023-08249-1

**Published:** 2023-04-26

**Authors:** Bunthen E, Ko Ko, Rattana Kim, Shintaro Nagashima, Serge Ouoba, Md Razeen Ashraf Hussain, Tomoki Sato, Channarena Chuon, Kanon Abe, Aya Sugiyama, Kazuaki Takahashi, Tomoyuki Akita, Rathavy Tung, Vichit Ork, Md. Shafiqul Hossain, Vonthanak Saphonn, Junko Tanaka

**Affiliations:** 1 Department of Epidemiology, Infectious Disease Control and Prevention, Graduate School of Biomedical and Health Sciences, Hiroshima University, 1-2-3, Kasumi, Minami-Ku, Hiroshima, 734-8551 Japan; 2 Payment Certification Agency (PCA), Ministry of Health, Phnom Penh, Cambodia; 3 National Maternal and Child Health Center (NMCHC), Ministry of Health, Phnom Penh, Cambodia; 4 Unité de Recherche Clinique de Nanoro (URCN), Institut de Recherche en Sciences de La Santé (IRSS), Nanoro, Burkina Faso; 5 Hiroshima City Funairi Citizens Hospital, Hiroshima, Japan; 6 Doctor Alliance of Union of Youth Federation of Cambodia (DAUYFC), Phnom Penh, Cambodia; 7 Ministry of Health, Phnom Penh, Cambodia; 8 National Immunization Program (NIP), Ministry of Health, Phnom Penh, Cambodia; 9 Expanded Program On Immunization, World Health Organization Country Office, Phnom Penh, Cambodia; 10 University of Health Sciences (UHS), Phnom Penh, Cambodia

**Keywords:** Epidemiology, Hepatitis B Virus, Mother-to-child transmission, Prevalence, Pregnant women, HBeAg, Longitudinal study

## Abstract

**Background:**

In countries with intermediate or high hepatitis B virus (HBV) endemicity, mother-to-child transmission (MTCT) represents the main route of chronic HBV infection. There is a paucity of information on HBV MTCT in Cambodia. This study aimed to investigate the prevalence of HBV infection among pregnant women and its MTCT rate in Siem Reap, Cambodia.

**Methods:**

This longitudinal study included two parts, study-1 to screen HBsAg among pregnant women and study-2 to follow up babies of all HBsAg-positive and one-fourth of HBsAg-negative mothers at their delivery and six-month post-partum. Serum or dried blood spot (DBS) samples were collected to examine HBV sero-markers by chemiluminescent enzyme immunoassay (CLEIA), and molecular analyses were performed on HBsAg-positive samples. Structured questionnaires and medical records were used to examine the risk factors for HBV infection. MTCT rate was calculated by HBsAg positivity of 6-month-old babies born to HBsAg-positive mothers and ascertained by the homology of HBV genomes in mother–child pair at 6-month-old.

**Results:**

A total of 1,565 pregnant women were screened, and HBsAg prevalence was 4.28% (67/1565). HBeAg positivity was 41.8% and was significantly associated with high viral load (*p* < 0.0001). Excluding subjects who dropped out due to restrictions during COVID-19, one out of 35 babies born to HBsAg-positive mothers tested positive for HBsAg at 6 months of age, despite receiving timely HepB birth dose and HBIG, followed by 3 doses of HepB vaccine. Hence the MTCT rate was 2.86%. The mother of the infected baby was positive for HBeAg and had a high HBV viral load (1.2 × 10^9^ copies/mL). HBV genome analysis showed 100% homology between the mother and the child.

**Conclusions:**

Our findings illustrate the intermediate endemicity of HBV infection among pregnant women in Siem Reap, Cambodia. Despite full HepB vaccination, a residual risk of HBV MTCT was observed. This finding supports the recently updated guidelines for the prevention of HBV MTCT in 2021, which integrated screening and antiviral prophylaxis for pregnant women at risk of HBV MTCT. Furthermore, we strongly recommend the urgent implementation of these guidelines nationwide to effectively combat HBV in Cambodia.

**Supplementary Information:**

The online version contains supplementary material available at 10.1186/s12879-023-08249-1.

## Background

Viral hepatitis remains one of the major global public health concerns and combating hepatitis has been stated in the Sustainable Development Goals (SDG 3.3). In 2016, the World Health Organization (WHO) called for the elimination of viral hepatitis as a public health threat by 2030. In 2019, the WHO estimated that 296 million individuals were chronically infected with hepatitis B virus (HBV) and about 820 000 people died from HBV infection [1]. HBV is most commonly transmitted from mother to child at birth (vertical transmission) as well as through horizontal transmission at the early age. Chronic infection is more likely in infants infected before the age of five [2]. Therefore, preventive measures against HBV infection at a young age would be more efficient.

HBV infection is highly endemic in most developing countries, including Cambodia. Aside from that, Cambodia has made significant progress in combating the virus. In 2005, the national immunization program (NIP) under Ministry of Health (MOH) of Cambodia included the hepatitis B (HepB) vaccine at birth and three follow-up doses (pentavalent vaccine including HepB at 6 weeks, 10 weeks, and 14 weeks of age). Since then, the vaccine coverage has been escalating yearly from 25% in 2007 to 90.2% in 2020 for HepB vaccine birth dose and from 80% in 2006 to 100% in 2020 for three pentavalent vaccine [3]. According to the 2017 nationwide study what we (Hiroshima University) had conducted in collaboration with NIP (Cambodia), WHO (Western Pacific Regional Office, WPRO), WHO (Cambodia), US CDC and University of Health Sciences (UHS, Cambodia), the hepatitis B surface antigen (HBsAg) prevalence was 0.56% in 5–7 years old children and 4.39% in their mothers (19–61 years old) [4]. As a result, the WHO WPRO recognized the achievement of the regional 2017 target of HBsAg prevalence < 1% in 5 years old children in Cambodia [5]. However, high prevalence in women of childbearing age showed the potential of vertical transmission, threatening the WHO target of eliminating hepatitis virus infection by 2030.

Following the abovementioned results from 2017 nationwide study, Cambodia needs to assess whether mother-to-child transmission of HBV still occurs or not. Therefore, this study aimed to investigate the prevalence of HBsAg among pregnant women and to assess the mother-to-child transmission (MTCT) of HBV in real-life practice in Siem Reap Province of Cambodia.

## Methods

### Sample size

The required sample size of HBsAg-positive mothers for investigating the MTCT of HBV was estimated to be at least 31 using the following formula under the assumption of a transmission rate of 2% and an absolute accuracy of 5% [6].

$$n={\left({z}_{1-\frac{\mathrm{\alpha }}{2}}\right)}^{2}\times \frac{p\left(1-p\right)}{{d}^{2}}$$

Where *n* = sample size, α = the significant level (0.05), Z _1-α/2_ = 1.96, *p* = 0.02, d = precision (0.05),

To get the minimum number of HBsAg-positive pregnant women, we expected to screen 1,500 pregnant women using the following assumptions: 4% of HBsAg prevalence among pregnant women in Cambodia, 65% participation rate, and 5% loss to follow-up rate.

### Study design and study subjects

This hospital-based prospective longitudinal study included two parts:

#### (Study-1) Screening of HBsAg among pregnant women to estimate the HBsAg prevalence among pregnant women

From February to September 2020, using the convenient sampling method, all pregnant women who visited one of three designated hospitals in Siem Reap Province, Cambodia (Mondule I Health Center, Angkor Chumm District Referral Hospital, and Siem Reap Provincial Referral Hospital) were tested for HBsAg using HBsAg rapid test (Abbott Determine™ HBsAg II Plus 100 T) after well explanation of study contents, its potential harms and benefits and receiving their informed consents voluntarily. We included all pregnant women irrespective of reproductive age, number of gravidities, parity, and month of gestation. The results from HBsAg rapid test were immediately given to pregnant women onsite with post-test counseling. Additionally, serum samples were collected from all participants for further analysis, and well-structured questionnaires (in local Khmer language) were used to collect their socio-demographic data and information about HBV infection. The status of other infectious diseases, such as syphilis and human immunodeficiency virus (HIV) infection were collected from the medical records.

#### (Study-2) Follow-up of their babies at delivery and at 6 months old to estimate the HBV MTCT

All HBsAg-positive pregnant women were recruited into the study-2, and their babies were followed up at delivery and 6 months post-partum. Furthermore, using a systematic random sampling method, every fourth of HBsAg-negative pregnant women were recruited for the study-2. Their babies were also followed up at delivery and 6 months post-partum to indirectly support the evidence of MTCT as the main transmission route in infants. If the eligible HBsAg-negative pregnant woman refused to participate, we recruited the subsequent HBsAg-negative woman as a substitution. We collected 4 ml of cord blood sample immediately after the delivery to examine the transplacental transfer of HBV seromarkers, 1 drop of peripheral blood (heel prick) for HBsAg rapid test (Abbott Determine™ HBsAg II Plus 100 T), and 3 drops for dried blood spots (DBS, HemaSpot™, Spot on Sciences, USA) in their 6-month follow-up visit for the post vaccination serological test to confirm the infection in baby which is in accordance with WHO guideline [7]. The delivery notes, HepB vaccine, and Hepatitis B Immune Globulin (HBIG) administration histories of all infants included in the study were collected from the medical records in each visit. The HBsAg rapid test results were immediately reported to the babies’ mothers/guardians with post-test counseling.

### Specimen storage and analysis

All collected serum and DBS samples were stored at -25℃ until analysis. The DBS extraction method was done as described in our previous study [8]. The HBsAg (Lumipulse HBsAg-HQ, Fujirebio Inc., Japan), Hepatitis B core antibody (anti-HBc: Lumi-pulse HBcAb-N, Fujirebio Inc., Japan), Hepatitis B surface antibody (anti-HBs: Lumi-pulse HBsAb-N, Fujirebio Inc., Japan), Hepatitis B e antigen (HBeAg: Lumi-pulse-I HBeAg, Fujirebio Inc., Japan), Hepatitis B e antibody (anti-HBe: Lumi-pulse HBeAb-N, Fujirebio Inc., Japan), and Hepatitis C antibody (anti-HCV: Lumi-pulse HCV, Fujirebio Inc., Japan) in both serum and DBS samples were detected using chemiluminescent enzyme immunoassay (CLEIA) as per the instructions of the manufacturer. The cut-off values for HBsAg, anti-HBs, anti-HBc, HBeAg and anti-HBe using DBS are 0.005 IU/mL, 3.4 mIU/mL, 0.6 COI, 1.0 COI and 45% respectively [9].

### Molecular measurement of HBV

All HBsAg-positive serum and DBS eluates (50 μL) were subjected to nucleic acid extraction using SMITEST EX-R&D (MBL, USA). The final pellet was diluted with 25 μL of RNase inhibitor-based water (Thermo Fisher Scientific, USA). For each sample, viral load was quantified by real-time polymerase chain reaction (Real Time-PCR, Thermo Fisher Scientific) using TaqMan™ Fast Advanced Master Mix (Applied Biosystem, Thermo Fisher Scientific, USA) and primers targeting HBV surface gene [10, 11] and the value was converted into copies per milliliter. All HBsAg-positive samples were undergone the amplification of partial genomes by nested PCR using TaKaRa Ex Taq® Hot Start version (Takara Bio. Inc, Shiga, Japan) and primer set targeting the overlapping region of surface and polymerase gene (nt475 to nt933). Those samples positive amplified to above region were then performed the amplification of full-length genome using the same protocol as described in previous study [10, 12]. Those samples negative amplified to above region, were then attempted for s-region fragment (partial genome sequence) using the primer set targeting surface gene (nt455-nt687) [10]. All positive amplified PCR products were directly sequenced as the same way mentioned in full length sequences [13]. The amplified products were subjected to sequencing using the Sanger method and the evolutionary analysis was done using the molecular evolutionary genetics analysis tool (MEGA X). The homology between mother and child HBV genomes was calculated using Genetyx Mac v21 (Genetyx Cooperation).

### Mutation analysis

The HBV full genomes were examined for mutations related to the potential vaccine or diagnostic escapes in the “a” determinant region at HBV surface antigen (nt121-nt149) or to HBeAg expression in the basal core promoter (BCP) (nt1753, nt1762 and nt1764) or precore (PC) (nt1814 and nt1896) regions, by direct visualization of genome sequences using Gentyx Mac v21 (Genetyx Cooperation).

### Data analysis

Data were managed and analyzed by statistical software STATA version 16.0 (STATA Corp LLC USA). The rate of MTCT was calculated as the number of newborns with HBsAg positive at six-month-old divided by the total number of infants born to HBsAg-positive mothers [7]. The proportions were estimated with their 95% confidence intervals. We employed univariable and multivariable logistic regression analysis to identify potential risk factors for HBsAg positivity, with age group, education level, and occupation of Household as independent variables. In addition, variables with a p-value < 0.2 in the univariable analysis were included in the multivariable model. A p-value of less than 0.05 was considered statistically significant.

## Results

### Characteristics of study participants

**Fig. 1:**
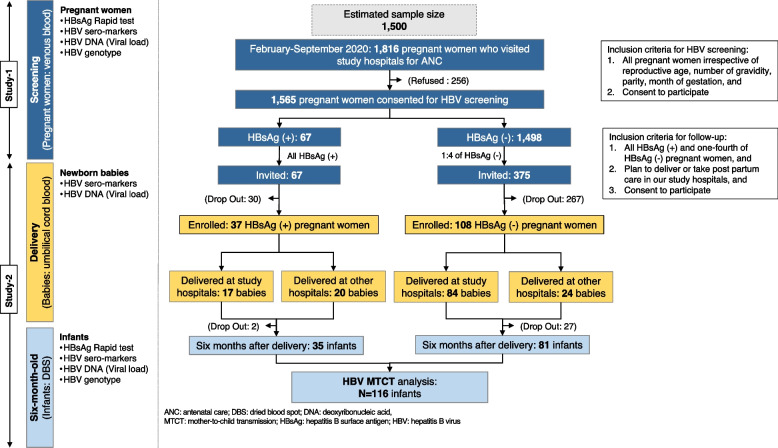
Flow chart of study subjects. This figure illustrates the flow of study subjects. 1,565 were screened, of whom 442 were invited for follow-up. There were only 145 subjects enrolled in follow-up. One hundred and one pregnant women delivered at the study hospitals, while another 44 pregnant women delivered in other hospitals. Finally, 116 infants were followed-up at six-month-old, of whom 35 infants were born to HBsAg positive mothers. ANC, antenatal care; HBsAg, hepatitis B surface antigen; HBV, hepatitis B virus; MTCT, mother-to-child transmission

During the recruitment period, 1,816 pregnant women visited the study hospitals for antenatal care and were eligible for HBV screening. Of them, 1,565 pregnant women participated in our study (study 1), resulting in a participation rate of 86.9%. We invited all HBsAg-positive (*n* = 67) and one-fourth of HBsAg-negative (*n* = 375) pregnant women to enroll in the follow-up study (study-2). However, due to restrictions during the COVID-19 pandemic, only 145 pregnant women could participate in the follow-up study, 37 of whom were HBsAg-positive. Finally, 116 six-month-old infants completed the follow-up until six-month-old, 35 of whom were born to HBsAg-positive mothers (Fig. 1).

The mean age of pregnant women at the screening was 28.3 ± 5.7 years. Almost all of them (95.5%, 1,495/1,565) were born before the introduction of HepB vaccine in the immunization program in Cambodia in 2001. History of blood transfusion, surgery, and HepB vaccine were reported by 2.43%, 13.04%, and 15.08% of participants, respectively. Regarding the background characteristics of HBsAg-positive and negative pregnant women, no significant difference was found except for the HepB vaccine status (*p* = 0.029) (Table 1). Multivariable analysis of HBsAg positivity indicated that pregnant women who ever received the HepB vaccine had significantly decreased odds of being positive for HBsAg (AOR: 0.22, 95% CI: 0.06–0.72, *p* = 0.011) (Table 2).

**Table 1:** Background characteristics of enrolled pregnant women in Siem Reap, Cambodia

Variables	Total (*N* = 1565)		HBsAg positive (*n* = 67)		HBsAg negative (*n* = 1498)		*p*-value
	Frequency	(%)	Frequency	(%)	Frequency	(%)	
Age	mean = 28.3 ± 5.7		mean = 29.4 ± 5.4		mean = 28.2 ± 5.7		0.098
15–19	70	4.47	0	0	70	4.67	0.420
20–24	338	21.60	15	22.39	323	21.56	
25–29	540	34.50	20	29.85	520	34.71	
30–34	391	24.98	20	29.85	371	24.77	
35–39	180	11.50	10	14.93	170	11.35	
≥ 40	46	2.94	2	2.99	44	2.94	
Education level							
≤ Primary School	324	20.7	13	19.4	311	20.76	0.758
High School	857	54.76	35	52.24	822	54.87	
University	384	24.54	19	28.36	365	24.37	
Occupation of Household							
Farmer/Fisherman/Laborer	255	16.29	6	8.96	249	16.62	0.318
Public Officer	217	13.87	10	14.93	207	13.82	
Privat Company Employee	495	31.63	26	38.81	469	31.31	
Self-Employed	598	38.21	25	37.31	573	38.25	
Number of children							
1–3	1469	93.87	63	94.03	1406	93.86	0.954
≥ 4	96	6.13	4	5.97	92	6.14	
Blood transfusion history							
No	1527	97.57	65	97.01	1462	97.60	0.762
Yes	38	2.43	2	2.99	36	2.40	
Surgical history							
No	1361	86.96	62	92.54	1299	86.72	0.166
Yes	204	13.04	5	7.46	199	13.28	
Ever received HepB							
Yes	236	15.08	3	4.48	233	15.55	0.029
No	1313	83.9	64	95.52	1249	83.38	
Don’t know	16	1.02	0	0	16	1.07

**Table 2:** Factors associated with HBsAg positivity amongst pregnant women in Siem Reap, Cambodia

Variables	Total (*N* = 1565)	HBsAg ( +)	Univariable analysis (*N* = 1565)			Multivariable analysis (*N* = 1506)		
		n (%)	OR	[95% CI]	*p*-value	AOR	[95% CI]	*p*-value
Age (mean = 28.3 ± 5.7)								
15–19	70	0 (0)	0	-	-	0	-	-
20–24	338	15 (4.44)	1	(Reference)	-	1	(Reference)	-
25–29	540	20 (3.70)	0.83	[0.42–1.64]	0.589	0.79	[0.39–1.58]	0.501
30–34	391	20 (5.12)	1.16	[0.58–2.30]	0.670	1.14	[0.57–2.28]	0.711
35–39	180	10 (5.56)	1.27	[0.55–2.88]	0.573	1.36	[0.59–3.19]	0.468
≥ 40	46	2 (4.35)	0.98	[0.22–4.42]	0.978	1.19	[0.25–5.61]	0.818
Education level								
≤ Primary School	324	13 (4.01)	1	(Reference)	-	1	(Reference)	-
High School	857	35 (4.08)	1.02	[0.53–1.95]	0.956	0.99	[0.49–1.98]	0.984
University	384	19 (4.95)	1.25	[0.61–2.56]	0.551	1.37	[0.61–3.03]	0.441
Occupation of Household								
Farmer/Fisherman/Laborer	255	6 (2.35)	1	(Reference)	-	1	(Reference)	-
Public Officer	217	10 (4.61)	2.00	[0.72–5.61]	0.185	2.18	[0.73–6.40]	0.158
Privat Company Employee	495	26 (5.25)	2.30	[0.93–5.66]	0.070	2.37	[0.92–6.11]	0.074
Self-Employed	598	25 (4.18)	1.81	[0.73–4.47]	0.198	1.92	[0.75–4.91]	0.171
Number of children								
1–3	1469	63 (4.29)	1	(Reference)	-	-	-	-
≥ 4	96	4 (4.17)	0.97	[0.35–2.72]	0.954	-	-	-
Blood transfusion history								
No	1527	65 (4.26)	1	(Reference)	-	-	-	-
Yes	38	2 (5.29)	1.25	[0.29–5.30]	0.763	-	-	-
Surgical history								
No	1361	62 (4.56)	1	(Reference)	-	1	(Reference)	-
Yes	204	5 (2.45)	0.53	[0.21–1.33]	0.173	0.5	[0.19–1.28]	0.152
Ever received HepB								
Yes	236	3 (1.27)	0.25	[0.08–0.81]	0.020	0.22	[0.06–0.72]	0.011
No	1313	64 (4.87)	1	(Reference)	-	1	(Reference)	-
Don’t know	16	0 (0)	0	-	-	-	-	-

Adjusted for: age, education level, occupation of Household, Surgical history, and ever received HBV vaccine

*OR* Odd ratio, *AOR* Adjusted odds ratio, *CI* Confident interval, *HBsAg* Hepatitis B surface antigen, *HepB* Hepatitis B vaccine

### Prevalence of HBV and HCV among pregnant women

**Fig. 2:**
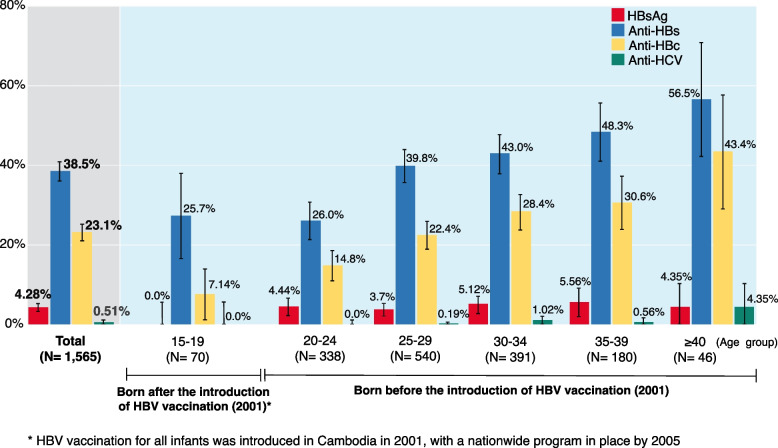
Distribution of HBV and HCV sero-markers among pregnant women in Siem Reap by age group. Among pregnant women in Siem Reap, the distribution of hepatitis B surface antigen was presented in Fig. 2. The red bar, blue bar, yellow bar, and green bar represent the prevalence of hepatitis B surface antigen, hepatitis B surface antibody, antibody to hepatitis b core antigen, and antibody to hepatitis C virus, respectively. Anti-HBc: antibody to hepatitis B core antigen; Anti-HBs: hepatitis B surface antibody Anti-HCV: antibody to hepatitis C virus; HBsAg: hepatitis B surface antigen

Out of 1,565 pregnant women, HBsAg was detected in 67, i.e., a prevalence of 4.28% (95% CI: 3.27–5.28%), none of whom was coinfected with syphilis nor HIV. The median age of HBsAg-positive pregnant women was 29 years (interquartile range [IQR]: 25–33). The prevalence of anti-HBs, anti-HBc and anti-HCV among pregnant women was 38.5% (95%CI: 36.1–40.9%), 23.1% (95%CI: 21.0–25.2%), and 0.51% (95%CI: 0.16–0.86%) respectively (Fig. 2). Among the 67 HBsAg-positive pregnant women, the prevalence of HBeAg and anti-HBe were 41.8% (95%CI: 30.0–53.6%) and 55.2% (95% CI:43.3–67.1%), respectively. HBeAg-positive pregnant women had a significantly higher viral load than HBeAg-negative pregnant women (*p* < 0.0001) (Supplementary Fig. 1a). The HBV viral load ranged from 0.2 × 10^2^ to 7.45 × 10^9^ copies/mL (median: 7.61 × 10^3^ copies/mL) and 28.4% of the HBsAg-positive pregnant women had a high viral load (≥ 10^6^ copies/mL). HBeAg-positivity was found to decrease with age (p for trend = 0.0299), but there was no significant difference in viral load between age groups (*p* = 0.7225) (Supplementary Fig. 1b).

### Mother-to-child transmission

**Fig. 3:**
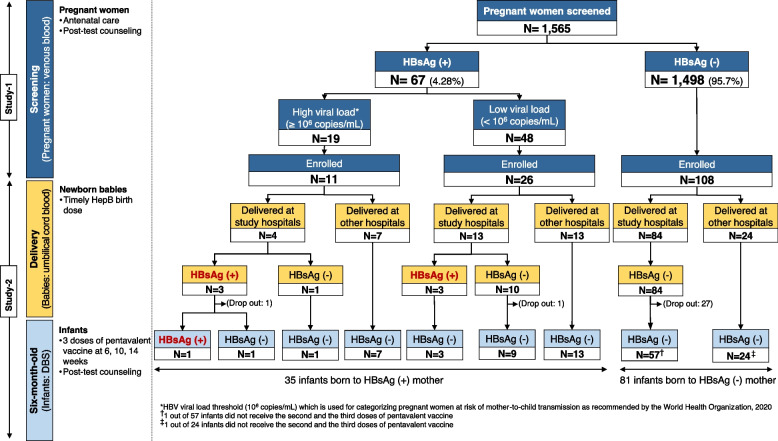
HBsAg status of infants at six-month-old stratified by maternal HBsAg and viral load. This figure illustrates the HBsAg of the newborn babies stratified by maternal HBsAg and viral load. HBsAg was detected in one baby born to HBsAg positive mother with high viral load, while none of those born to HBsAg negative mother was detected. ANC: antenatal care; DBS: dried blood spot; HBsAg: hepatitis B surface antigen; HBV: hepatitis B virus; HepB: hepatitis B vaccine

Of the 145 pregnant women who participated in study-2, 111 (including 17 HBsAg positive) delivered in our study hospitals, and cord blood samples were collected. HBsAg was identified in 6 umbilical cord blood samples, 3 of which had detected HBV viral load ranging from 1.6 × 10^2^ to 1.5 × 10^6^ copies/mL (Fig. 3 and Table 3).

**Table 3:** Characteristics of 6 infants who had HBsAg in their umbilical cord blood

Cases	Mothers (Venous Blood)			At delivery (umbilical Cord Blood)				Six-month-old infants (DBS)							
	Age (year)	Viral load (copies/mL)	HBeAg	HBsAg	Viral load (copies/mL)	HBeAg	Anti-HBc	Sex	Vaccination^a^	HBIG received	HBsAg	Viral load (copies/mL)	HBeAg	Anti-HBc	Anti-HBs
1	30	1.2 × 10^9^	( +)	( +)	1.6 × 10^6^	( +)	( +)	Female	Completed	Yes	( +)	1.8 × 10^9^	( +)	( +)	(-)
2	29	6.7 × 10^7^	( +)	( +)	8.1 × 10^4^	( +)	( +)	Male	Completed	No	(-)	N/A	N/A	(-)	( +)
3	33	9.3 × 10^5^	( +)	( +)	1.6 × 10^2^	( +)	( +)	Female	Completed	No	(-)	N/A	N/A	(-)	( +)
4	33	2.3 × 10^4^	(-)	( +)	Undetermined	(-)	( +)	Female	Completed	No	(-)	N/A	N/A	(-)	(-)
5	24	2.7 × 10^7^	( +)	( +)	Undetermined	( +)	( +)	Female	Lost to Follow-up						
6	30	2 × 10^3^	( +)	( +)	Undetermined	( +)	( +)	Male	Completed	No	(-)	N/A	N/A	(-)	( +)

*N/A* Not applicable

^a^Hepatitis B vaccination at birth within 24 h and pentavalent doses at 6 weeks, 10 weeks, and 14 weeks of age

**Fig. 4:**
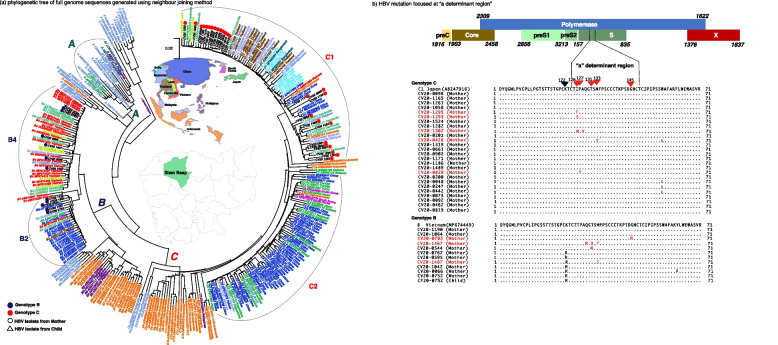
Using reference strains from GenBank and 38 isolates (included mother and child pair) of our study **a**) Phylogenetic tree of full genome sequences generated using neighbour-joining method **b**) Mutation focused at “a” determinant region. **a** Using neighbour-joining method, displays sub-genotype of the full genome sequence of 38 isolates of our study, including one pair of a child and her mother. Blue circles and red circles represent genotype B and genotype C of the isolates from our study, respectively. The HBsAg positive child (CF20-0752 with blue triangle) and her mother (CV20-0752 with blue circle) were identified having the same sequence, confirming the HBV mother-to-child transmission. **b** shows the mutation at the “a” determinant region using 38 isolates from our study. Mutation at the “a” determinant was identified in 9 isolates (24.3%). No mutation at the “a” determinant region was detected in the pair of HBsAg-positive mother and child

The 6-month post-partum visit was performed in 116 infants, of whom 35 were born to HBsAg-positive mothers. One of these 35 infants tested positive for HBsAg, with a viral load of 1.8 × 10^9^ copies/mL, whereas none of the 81 infants born to HBsAg-negative mothers tested positive, resulting in a MTCT rate of 2.86% (95% CI: 0.50–14.53%). Three of the 35 infants born to HBsAg-positive mothers received HBIG at birth, amongst which two infants were born at other hospitals. Regarding HepB vaccination, 100% (116/116) and 98.3% (114/116) of the infants received the HepB birth dose vaccine and three follow-up doses (pentavalent vaccine), respectively. Only two infants had incomplete HepB vaccination, and one infant was born in another hospital. The infected infant was born to a 30-year-old mother who was HBeAg-positive with a high HBV viral load (1.2 × 10^9^ copies/mL) (Fig. 3 and Supplementary Fig. 2). By medical records, this pregnant woman was not exposed to any risk for MTCT of HBV, such as invasive procedure (amniocentesis, etc.). The delivery occurred at 39 weeks of gestation by on-demand cesarian section. HBsAg and HBeAg were detected in the cord blood, which HBV viral load was 1.6 × 10^6^ copies/mL. The infant received HepB vaccine birth dose and HBIG within 24 h and completed a three-dose pentavalent vaccine. The mother of the infected child did not receive antiviral prophylaxis during her pregnancy. The whole-genome sequencing was successfully achieved from the pair of HBsAg-positive child (CF20-0752) and her mother (CV20-0752). Both belonged to genotype B4. The phylogenetic analysis revealed a 100% homology sequence in the whole genome (Fig. 4a). Moreover, mutation at the “a” determinant region of the positive pair was not detected (Fig. 4b).

### Genotype distribution and mutation analyses

Out of 67 HBsAg-positive samples from pregnant women, partial and full genome sequences were successfully performed in 24 and 37 (Fig. 4a) samples, respectively, and genotypes were identified in 61 samples. HBV genotype C was predominant (42/61, 68.9%), followed by B (19/61, 31.1%). Full genome sequence analysis of 37 HBV isolates found a mutation rate at the “a” determinant region of the surface protein of 24.3% (9/37) (Fig. 4b). Additionally, 17 HBV isolates had mutations in the BPC and/or PC regions (11 had BCP mutations, 4 had PC mutations and 2 had combined BCP and PC mutations). Among them, 6 isolates (35.3%) were positive for HBeAg and had high viral loads, whereas 11 isolates (64.7%) were HBeAg-negative. Despite HBeAg negativity, one sample (9%) showed a high viral load > 10^6^ copies/mL.

## Discussion

This prospective longitudinal study was conducted among pregnant women and their newborn babies in Siem Reap, Cambodia. We determined the prevalence of HBsAg among pregnant women and evaluated the MTCT of the virus in the real-life practice of the current Cambodian health care system. In our study, HBsAg was detected in 4.28% (95% CI: 3.27%-5.28%) of pregnant women, which is consistent with the previous findings [4]. But it is lower than the recent report from Segeral et al. (6.2%) [14]. This HBsAg-positive rate indicates intermediate endemicity of HBV infection among pregnant women in Cambodia. Nevertheless, our result is lower than those of neighboring countries where HBsAg prevalence among pregnant women were reported as 10.8%-12.6% in Vietnam, 5.44%-8.0% in Lao PDR, and 6.2%-8.3% in Thailand [15–21].

Both horizontal and vertical transmission routes exist in countries with intermediate and high endemicity of HBV, but vertical transmission is the most common [22]. Therefore, our main concern was how much MTCT was occurring in Cambodia to evaluate the potential threat of MTCT, a significant risk factor for chronic HBV infection [23].

The WHO recommends all countries to include the HepB vaccine in their national immunization programs. As a result, the prevalence of HBV infection has decreased globally in many countries. In Cambodia, the most recent report (as of 2020) about HepB vaccine coverage was 78.4%-90.2% for timely birth dose (within 24 h) and 88.7%-100% for the three-dose pentavalent vaccine [3, 4]. In our study, 100% of the infants received a timely birth dose, and 98.3% received the three-dose pentavalent vaccine. Our higher HepB vaccine coverage could be due to a study effect, as pregnant women who participated were given information about HBV and were prompted to vaccinate their children. Although a cornerstone of HBV prevention, vaccination alone might not be enough to achieve the WHO target of 0.1% HBsAg prevalence in children by 2030﻿ [24, 25]. Moreover, the HepB vaccine may not be effective in preventing intrauterine infection, which occurs when the virus is transmitted from the mother to the fetus during pregnancy [26, 27].

HBV intrauterine infection is defined by the presence of HBsAg and/or HBV DNA in the umbilical cord blood and has been reported to be between 5.36% and 40% [28–31]. In our study, six of 17 cord blood samples collected at delivery tested positive for HBsAg. Five infants could be assessed at 6 months post-partum for HBV infection, and one of them was still positive for both HBsAg and anti-HBc, with a high viral load (1.8 × 10^9^ copies/mL), despite receiving HepB vaccine and HBIG at birth. The homology analysis of HBV full genome extracted from this mother–child pair was 100%, suggesting that the virions of this infant were more likely derived from her mother during pregnancy. We did not find mutations in the “a” determinant region of the HBV surface gene, which is reportedly associated with vaccine or diagnostic escapes. This finding supports that intrauterine infection can be a cause of immunoprophylaxis failure [32, 33].

The risk of intrauterine infections can be reduced if the mother receives antiviral therapy during pregnancy [24, 34], prompting the WHO to recommend the administration of tenofovir in the third trimester of pregnancy until at least birth in pregnant women with high viral load, i.e.load 200,000 IU/mL (equivalent to 10^6^ copies/mL) [35]. In resource-limited countries, WHO recommends using HBeAg as an alternative to viral quantification. In our study, HBeAg-positive women had a significantly higher viral load than the HBeAg-negative women, which is coherent with other studies [36–38], and supports using HBeAg as an alternative for antiviral treatment eligibility. However, HBeAg-negative pregnant women may have a high viral load in some cases. Mutations in the BPC or PC have been shown to reduce the expression of HBeAg [39, 40], and these pregnant women may have high viral loads, thus a high risk of transmission. In our study, only one HBeAg-negative pregnant woman had a high HBV viral load, above 10^6^ copies/mL. Full sequence analysis found mutations in the BCP region, consistent with a lack of HBeAg expression. This finding suggests caution in using HBeAg positivity as an eligible criterion for antiviral prophylaxis, especially in areas where HBV viral load measurement is limited. This finding also calls for further molecular studies to assess the burden of these mutations in HBsAg-positive and HBeAg-negative pregnant women to better guide MTCT prevention strategies in Cambodia.

In our study, the infant infected with HBV received a timely HepB vaccine birth dose, HBIG, and three follow-up pentavalent vaccine doses. Several studies have also reported that a residual risk of MTCT at 3%-7% despite timely vaccination [25, 41–43]. The mother had a high viral load (≥ 10^6^ copies/mL) and was HBeAg positive but did not receive antiviral prophylaxis. Treatment with tenofovir during the third trimester of pregnancy could have prevented the transmission of HBV from the mother to her infant. At the time of the study, antiviral prophylaxis was not recommended in Cambodia. In 2021, Cambodia established guidelines for preventing MTCT of HBV, which were adopted from the WHO 2020 recommendations. But as of 2022, these guidelines were not implemented nationwide, only in the urban capital city. Hence, vaccination at birth followed by three booster doses have been the only interventions used to reduce the MTCT of HBV, as HBIG is optional if available and affordable. Therefore, we urge nationwide implementation of the recently updated guidelines, adding antiviral prophylaxis to the current prevention measures, to achieve the WHO target of 0.01% HBsAg prevalence in children by 2030 in Cambodia. Immediate nationwide implementation of the guidelines can be challenging for Cambodia because of limited funding, low awareness, stigma, discrimination, and limited access to health care services. Therefore, sustained effort and collaboration from all the stakeholders are required to reach this important target.

This study has limitations. There was a high drop-out rate in the follow-up study, and the infection status of these infants is unknown. Therefore, over or under-estimation of MTCT in our study cannot be ruled out. However, the causes of drop-out were mainly due to restrictions during the COVID-19 pandemic making it less likely to affect the outcome of the study. Moreover, the background characteristics of those who completed follow-up and those who did not were not significantly different (Supplementary Table). To the best of our knowledge, this is the first observational study on HBV MTCT in the real-life practice of the current Cambodian healthcare system, and the data provided are valuable for health policy making.

## Conclusions

Our findings illustrate intermediate endemicity of HBV infection among pregnant women in Siem Reap, Cambodia. Considering a residual risk of HBV MTCT despite full HepB vaccination, implementing the recently updated (2021) national guidelines for preventing HBV MTCT, which integrated screening and antiviral prophylaxis for pregnant women at high risk, is urgently required. To overcome the challenges and to get complete combating HBV infection in Cambodia, sustained effort and collaboration from all the stakeholders with a multidisciplinary approach are strongly recommended.

## Supplementary Information

**Additional file 1: Supplementary Figure 1.** Distribution of (a) HBV viral load by HBeAg and (b) HBV viral load and HBeAg by age group among the 67 HBsAg positive pregnant women

**Additional file 2: Supplementary Figure 2.** HBsAg status of infants at six-month-old stratified by maternal HBsAg and HBeAg.

**Additional file 3:** **Supplementary Table.** Comparison backgroundcharacteristics between followed-up and drop-out pregnant women in study-2.

## Data Availability

The datasets generated and/or analyzed during the current study are available in the GenBank/DDBJ (DNA Data Bank of Japan) repository with the accession numbers from LC753640 to LC753677 (HBV full genome) and LC753678 to LC753701 (HBV partial genome).

## Abbreviations

Anti HBs: Hepatitis B surface antibody
Anti-HBc: Hepatitis B core antibody
Anti-HCV: Hepatitis C antibody
AOR: Adjusted odds ratio
BPC: Basal core promotor;
BD: Birth dose
CI: Confident interval
CLEIA: Chemiluminescent enzyme immunoassay
DBS: Dried Blood Spot
HBIG: Hepatitis B Immune Globulin
HBsAg: Hepatitis B surface antigen
HBV: Hepatitis B virus
HepB: Hepatitis B vaccine
HIV: Human immunodeficiency virus
IQR: Interquartile range
MTCT: Mother-to-child transmission
n.s: Not significant
PC: Precore
SDG: Sustainable Development Goals
WHO: World Health Organization
