# Supplementary material for: Residual risk of mother-to-child transmission of HBV despite timely Hepatitis B vaccination: a major challenge to eliminate hepatitis B infection in Cambodia
Source: BMC Infect Dis. 2023 Apr 26;23:261. doi: 10.1186/s12879-023-08249-1 (PMC10131410; doi:10.1186/s12879-023-08249-1)
Supplement: Supplementary file 1 — Additional file 1: Supplementary Figure 1. Distribution of (a) HBV viral load by HBeAg and (b) HBV viral load and HBeAg by age group among the 67 HBsAg positive pregnant women [file 12879_2023_8249_MOESM1_ESM.pdf]

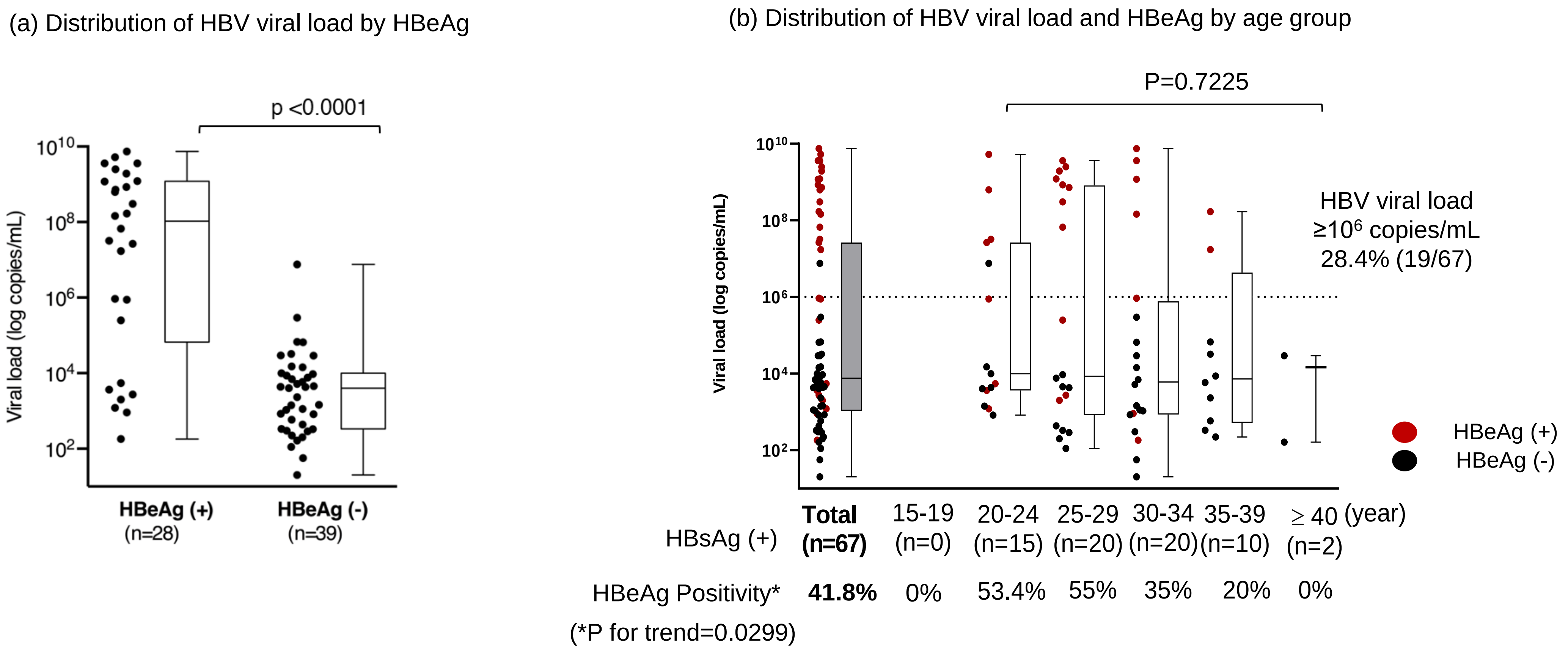

Supplementary Figure 1: Distribution of (a) HBV viral load by HBeAg and (b) HBV viral load and HBeAg by age group among the 67 HBsAg positive pregnant women
