# Supplementary material for: Residual risk of mother-to-child transmission of HBV despite timely Hepatitis B vaccination: a major challenge to eliminate hepatitis B infection in Cambodia
Source: BMC Infect Dis. 2023 Apr 26;23:261. doi: 10.1186/s12879-023-08249-1 (PMC10131410; doi:10.1186/s12879-023-08249-1)
Supplement: Supplementary file 2 — Additional file 2: Supplementary Figure 2. HBsAg status of infants at six-month-old stratified by maternal HBsAg and HBeAg. [file 12879_2023_8249_MOESM2_ESM.pdf]

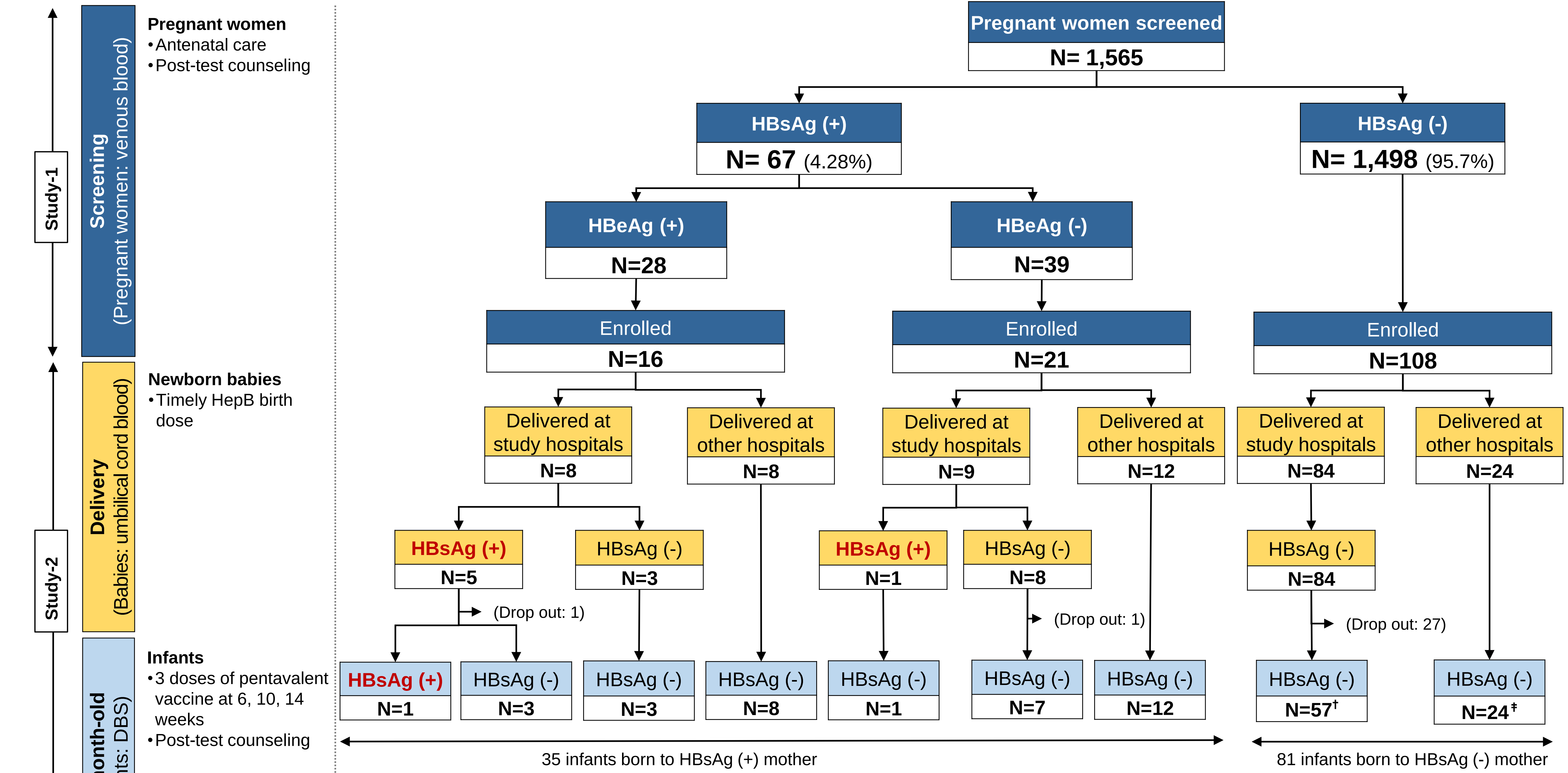

†1 out of 57 infants did not receive the second and the third doses of pentavalent vaccine
‡1 out of 24 infants did not receive the second and the third doses of pentavalent vaccine

Supplementary Figure 2: HBsAg status of infants at six-month-old stratified by maternal HBsAg and HBeAg
