# Supplementary material for: Residual risk of mother-to-child transmission of HBV despite timely Hepatitis B vaccination: a major challenge to eliminate hepatitis B infection in Cambodia
Source: BMC Infect Dis. 2023 Apr 26;23:261. doi: 10.1186/s12879-023-08249-1 (PMC10131410; doi:10.1186/s12879-023-08249-1)
Supplement: Supplementary file 3 — Additional file 3: Supplementary Table. Comparison backgroundcharacteristics between followed-up and drop-out pregnant women in study-2. [file 12879_2023_8249_MOESM3_ESM.docx]

**Supplementary Table:** Comparison background characteristics between followed-up and drop-out pregnant women in study-2

| Variables | | Appointed for study-2 (N=442) | | p-value |
| --- | --- | --- | --- | --- |
|  |  | Followed-up n=145 (%) | Drop out  n=297 (%) |  |
| Age (mean=27.9 ± 5.5) | |  |  |  |
|  | 15-19 | 3 (2.1) | 16 (5.4) | 0.137 |
|  | 20-24 | 29 (20.0) | 80 (29.9) |  |
|  | 25-29 | 59 (40.6) | 97 (32.7) |  |
|  | 30-34 | 32 (22.1) | 69 (23.2) |  |
|  | 35-39 | 19 (13.1) | 26 (8.8) |  |
|  | ≥40 | 3 (2.1) | 9 (3.0) |  |
| Education level |  |  |  |  |
|  | ≤Primary School | 32 (22.1) | 63 (21.2) | 0.068 |
|  | High School | 65 (44.8) | 164 (55.2) |  |
|  | University | 48 (33.1) | 70 (23.6) |  |
| Occupation |  |  |  |  |
|  | Agricultural/Fishery/Laborer | 24 (16.6) | 47 (15.8) | 0.645 |
|  | Public Officer | 22 (15.2) | 41 (13.8) |  |
|  | Privat Company Employee | 35 (24.1) | 89 (30.0) |  |
|  | Self-Employed | 64 (44.1) | 120 (40.4) |  |
| Number of children | |  |  |  |
|  | 1-3 | 136 (93.8) | 283 (95.3) | 0.507 |
|  | ≥4 | 9 (6.2) | 14 (4.7) |  |
| Blood transfusion history | |  |  |  |
|  | No | 140 (96.5) | 292 (98.3) | 0.241 |
|  | Yes | 5 (3.5) | 5 (1.7) |  |
| Surgical history |  |  |  |  |
|  | No | 129 (89.0) | 256 (86.2) | 0.415 |
|  | Yes | 16 (11.0) | 41 (13.8) |  |
| Ever received HepB | |  |  |  |
|  | No/ Don’t know | 117 (80.7) | 253 (85.2) | 0.229 |
|  | Yes | 28 (19.3) | 44 (14.8) |  |
